# Supplementary material for: Does hippocampal volume explain performance differences on hippocampal-dependant tasks?
Source: Neuroimage. 2020 Nov 1;221:117211. doi: 10.1016/j.neuroimage.2020.117211 (PMC7762813; doi:10.1016/j.neuroimage.2020.117211)
Supplement: Supplementary file 1 [file mmc1.docx]

**Clark et al. Supplementary Materials**

**Supplementary Methods**

**Table S1.** Double scoring of the scene construction task.

|  | | Rating | | | | |
| --- | --- | --- | --- | --- | --- | --- |
|  | Spatial References | | Entities Present | Sensory Descriptions | Thoughts/  Emotions/Actions | Quality Ratings |
| **For each individual scene** | | | | | | |
|  |  | |  |  |  |  |
| n = 308 | 0.90 | | 0.96 | 0.94 | 0.90 | 0.90 |
|  |  | |  |  |  |  |
| **For each individual participant (i.e. score is averaged across the seven scenes)** | | | | | | |
|  | | | | | | |
| n = 44 | 0.91 | | 0.99 | 0.97 | 0.91 | 0.93 |
|  |  | |  |  |  |  |

*Note.* Inter-class correlation coefficients from a two way random effect model looking for absolute agreement for each content score and for the quality ratings. Four experimenters scored the whole data set (n = 217 participants, 1519 individual scenes) with double scoring performed on 20% of the data (n = 44 participants, 308 scenes) proportionally for each original experimenter.

**Table S2.** Double scoring of the autobiographical interview.

|  | | Rating | | | | | | |
| --- | --- | --- | --- | --- | --- | --- | --- | --- |
|  | Internal Event | | Internal Place | Internal Time | Internal Perceptual | Internal Emotion | Internal Sum | External Sum |
| **For each individual memory** | | | | | | |  |  |
|  |  | |  |  |  |  |  |  |
| n = 215 | 0.92 | | 0.85 | 0.94 | 0.92 | 0.86 | 0.94 | 0.84 |
|  |  | |  |  |  |  |  |  |
| **For each individual participant (i.e. score is averaged across the five memories)** | | | | | | | | |
|  | | | | | | |  |  |
| n = 43 | 0.95 | | 0.88 | 0.96 | 0.94 | 0.81 | 0.97 | 0.87 |
|  |  | |  |  |  |  |  |  |

*Note.* Inter-class correlation coefficients from a two way random effects model looking for absolute agreement for each score on the autobiographical interview. Three experimenters scored the whole data set (n = 217 participants, 1085 individual memories) and double scoring was performed 20% of the data (n = 43 participants, 215 individual memories) proportionally for each original experimenter.

**Table S3.** Double scoring of the future thinking task.

|  | | Rating | | | | |
| --- | --- | --- | --- | --- | --- | --- |
|  | Spatial References | | Entities Present | Sensory Descriptions | Thoughts/  Emotions/Actions | Quality Ratings |
| **For each individual scene** | | | | | | |
|  |  | |  |  |  |  |
| n = 132 | 0.90 | | 0.94 | 0.93 | 0.88 | 0.90 |
|  |  | |  |  |  |  |
| **For each individual participant (i.e. score is averaged across the three future scenes)** | | | | | | |
|  | | | | | | |
| n = 44 | 0.94 | | 0.95 | 0.96 | 0.88 | 0.92 |
|  |  | |  |  |  |  |

*Note.* Inter-class correlation coefficients from a two way random effects model looking for absolute agreement for each content score and for the quality ratings. Four experimenters scored the whole data set (n = 217 participants, 651 individual future scenes) with double scoring performed on 20% of the data (n = 44 participants, 132 future scenes) proportionally for each original experimenter.

**Table S4.** Double scoring of the navigation sketch maps.

|  | Rating | | | | | | |
| --- | --- | --- | --- | --- | --- | --- | --- |
|  | | Road Segments | Road Junctions | Number of Landmarks | Landmark Placement | Map Orientation | Map Categorisation |
|  | |  |  |  |  |  |  |
| n = 42 | | 0.95 | 0.96 | 0.97 | 0.96 | 0.96 | 0.89 |
|  | |  |  |  |  |  |  |

*Note.* Inter-class correlation coefficients from a two way random effects model looking for absolute agreement for each score on the navigation sketch maps. Three experimenters scored the whole data set (n = 217) and double scoring was performed on 20% of the data (n = 42 participants) proportionally for each original experimenter.

**Supplementary Results**

**Primary VBM analyses: Results outside of the hippocampus**

Only one association was identified between cognitive task performance and grey matter volume outside of the hippocampus (when using a statistical threshold of p < 0.05 FWE whole brain corrected). A negative association was observed between 22 voxels in the right parahippocampal cortex and AI vividness ratings (peak coordinates = 24 -23 -28, peak t = 4.78, p _FWE whole brain corrected_ = 0.025).

**Table S5.** Partial correlations between task performance and hippocampal grey matter volume with age, gender, total intracranial volume and MRI scanner as covariates.

| Performance variable | Whole hippocampus | | Anterior hippocampus | | Posterior hippocampus | | Posterior/Anterior hippocampus ratio | | |  |
| --- | --- | --- | --- | --- | --- | --- | --- | --- | --- | --- |
|  | r | p | r | p | r | p | | r | p | |
|  |  |  |  |  |  |  | |  |  | |
| **Scene construction** | | | | | | | | | | |
| Experiential index | 0.054 | 0.89 | 0.061 | 0.89 | 0.037 | 0.89 | | -0.021 | 0.89 | |
| Spatial references | 0.082 | 0.89 | 0.079 | 0.89 | 0.068 | 0.89 | | 0.00 | 1.00 | |
| Entities present | -0.013 | 0.89 | -0.062 | 0.89 | 0.034 | 0.89 | | 0.10 | 0.89 | |
| Sensory descriptions | 0.023 | 0.89 | 0.069 | 0.89 | -0.024 | 0.89 | | -0.095 | 0.89 | |
| Thoughts/emotions/actions | -0.043 | 0.89 | -0.066 | 0.89 | -0.013 | 0.89 | | 0.037 | 0.89 | |
| Spatial coherence index | 0.029 | 0.89 | 0.033 | 0.89 | 0.018 | 0.89 | | -0.021 | 0.89 | |
|  |  |  |  |  |  |  | |  |  | |
| **Autobiographical interview** | | | | | | | | | | |
| Internal details | -0.054 | 0.87 | -0.004 | 0.95 | -0.089 | 0.71 | | -0.11 | 0.69 | |
| External details | -0.013 | 0.92 | -0.067 | 0.87 | 0.039 | 0.87 | | 0.10 | 0.69 | |
| Internal events | -0.069 | 0.87 | -0.014 | 0.92 | -0.11 | 0.69 | | -0.11 | 0.69 | |
| Internal time | -0.027 | 0.87 | -0.025 | 0.87 | -0.024 | 0.87 | | -0.013 | 0.92 | |
| Internal place | -0.054 | 0.87 | -0.044 | 0.87 | -0.053 | 0.87 | | -0.031 | 0.87 | |
| Internal perceptual | -0.005 | 0.95 | 0.036 | 0.87 | -0.042 | 0.87 | | -0.091 | 0.71 | |
| Internal thoughts/emotions | -0.033 | 0.87 | -0.027 | 0.87 | -0.032 | 0.87 | | -0.027 | 0.87 | |
| Vividness rating | -0.13 | 0.69 | -0.095 | 0.71 | -0.14 | 0.69 | | -0.072 | 0.87 | |
|  |  |  |  |  |  |  | |  |  | |
| **Future thinking** | | | | | | | | | | |
| Experiential index | 0.029 | 0.89 | 0.064 | 0.89 | -0.010 | 0.89 | | -0.075 | 0.89 | |
| Spatial references | 0.068 | 0.89 | 0.11 | 0.89 | 0.020 | 0.89 | | -0.071 | 0.89 | |
| Entities present | -0.021 | 0.89 | -0.017 | 0.89 | -0.021 | 0.89 | | -0.010 | 0.89 | |
| Sensory descriptions | -0.016 | 0.89 | 0.026 | 0.89 | -0.051 | 0.89 | | -0.083 | 0.89 | |
| Thoughts/emotions/actions | -0.034 | 0.89 | -0.051 | 0.89 | -0.011 | 0.89 | | 0.025 | 0.89 | |
| Spatial coherence index | -0.025 | 0.89 | -0.011 | 0.89 | -0.031 | 0.89 | | -0.037 | 0.89 | |
|  |  |  |  |  |  |  | |  |  | |
| **Navigation** | | | | | | | | | | |
| Overall navigation score | 0.071 | 0.55 | 0.12 | 0.36 | 0.015 | 0.91 | | -0.10 | 0.36 | |
| Movie clip recognition | 0.11 | 0.36 | 0.057 | 0.62 | 0.14 | 0.36 | | 0.10 | 0.36 | |
| Scene recognition | -0.18 | 0.91 | -0.016 | 0.91 | -0.016 | 0.91 | | -0.002 | 0.98 | |
| Proximity judgements | 0.082 | 0.46 | 0.060 | 0.61 | 0.084 | 0.46 | | 0.030 | 0.88 | |
| Route knowledge | 0.11 | 0.36 | 0.16 | 0.36 | 0.042 | 0.78 | | -0.11 | 0.36 | |
| Sketch map | 0.060 | 0.61 | 0.11 | 0.36 | 0.004 | 0.98 | | -0.10 | 0.36 | |
|  |  |  |  |  |  |  | |  |  | |

Note. P values are Benjamini-Hochberg false discovery rate corrected at p < 0.05.

**Table S6.** Partial correlations between task performance and hippocampal grey matter volume in the male participants with age, total intracranial volume and MRI scanner as covariates.

| Performance variable | Whole hippocampus | | Anterior hippocampus | | Posterior hippocampus | | Posterior/Anterior hippocampus ratio | | |  |
| --- | --- | --- | --- | --- | --- | --- | --- | --- | --- | --- |
|  | r | p | r | p | r | p | | r | p | |
|  |  |  |  |  |  |  | |  |  | |
| **Scene construction** | | | | | | | | | | |
| Experiential index | -0.054 | 0.83 | -0.073 | 0.83 | -0.023 | 0.89 | | 0.056 | 0.83 | |
| Spatial references | -0.08 | 0.83 | -0.083 | 0.83 | -0.059 | 0.83 | | 0.025 | 0.89 | |
| Entities present | -0.11 | 0.78 | -0.17 | 0.59 | -0.032 | 0.89 | | 0.14 | 0.72 | |
| Sensory descriptions | -0.12 | 0.78 | -0.049 | 0.83 | 0.16 | 0.59 | | -0.13 | 0.72 | |
| Thoughts/emotions/actions | -0.1 | 0.83 | -0.17 | 0.59 | -0.006 | 0.95 | | 0.17 | 0.59 | |
| Spatial coherence index | 0.034 | 0.89 | 0.006 | 0.95 | 0.053 | 0.83 | | 0.058 | 0.83 | |
|  |  |  |  |  |  |  | |  |  | |
| **Autobiographical interview** | | | | | | | | | | |
| Internal details | -0.086 | 0.79 | -0.039 | 0.84 | -0.11 | 0.79 | | -0.10 | 0.79 | |
| External details | -0.037 | 0.84 | -0.080 | 0.79 | 0.015 | 0.91 | | 0.090 | 0.79 | |
| Internal events | -0.038 | 0.84 | 0.022 | 0.90 | -0.088 | 0.79 | | -0.12 | 0.79 | |
| Internal time | -0.12 | 0.79 | -0.12 | 0.79 | -0.096 | 0.79 | | 0.010 | 0.92 | |
| Internal place | -0.081 | 0.79 | -0.055 | 0.82 | -0.089 | 0.79 | | -0.071 | 0.82 | |
| Internal perceptual | -0.11 | 0.79 | -0.053 | 0.82 | -0.14 | 0.79 | | -0.12 | 0.79 | |
| Internal thoughts/emotions | -0.024 | 0.90 | -0.063 | 0.82 | 0.02 | 0.90 | | 0.068 | 0.82 | |
| Vividness rating | -0.079 | 0.79 | -0.10 | 0.79 | -0.036 | 0.84 | | 0.060 | 0.82 | |
|  |  |  |  |  |  |  | |  |  | |
| **Future thinking** | | | | | | | | | | |
| Experiential index | -0.12 | 0.52 | -0.056 | 0.68 | -0.15 | 0.52 | | -0.11 | 0.52 | |
| Spatial references | -0.12 | 0.52 | -0.013 | 0.90 | -0.19 | 0.52 | | -0.20 | 0.52 | |
| Entities present | -0.11 | 0.52 | -0.094 | 0.55 | -0.11 | 0.52 | | -0.024 | 0.87 | |
| Sensory descriptions | -0.092 | 0.55 | -0.041 | 0.78 | -0.12 | 0.52 | | -0.087 | 0.55 | |
| Thoughts/emotions/actions | -0.12 | 0.52 | -0.14 | 0.52 | -0.066 | 0.67 | | 0.062 | 0.67 | |
| Spatial coherence index | -0.11 | 0.52 | -0.11 | 0.52 | -0.085 | 0.55 | | 0.021 | 0.87 | |
|  |  |  |  |  |  |  | |  |  | |
| **Navigation** | | | | | | | | | | |
| Overall navigation score | 0.07 | 0.77 | 0.13 | 0.46 | -0.005 | 0.97 | | -0.14 | 0.43 | |
| Movie clip recognition | 0.24 | 0.09 | 0.16 | 0.38 | 0.27 | 0.072 | | 0.15 | 0.41 | |
| Scene recognition | 0.12 | 0.48 | 0.084 | 0.67 | 0.12 | 0.48 | | 0.061 | 0.81 | |
| Proximity judgements | 0.012 | 0.97 | 0.017 | 0.97 | 0.004 | 0.97 | | -0.011 | 0.97 | |
| Route knowledge | 0.18 | 0.35 | 0.27 | 0.072 | 0.044 | 0.88 | | -0.24 | 0.090 | |
| Sketch map | 0.044 | 0.88 | 0.10 | 0.55 | -0.025 | 0.97 | | -0.14 | 0.43 | |
|  |  |  |  |  |  |  | |  |  | |

Note. P values are Benjamini-Hochberg false discovery rate corrected at p < 0.05.

**Table S7.** Partial correlations between tasks performance and hippocampal grey matter volume in the female participants with age, total intracranial volume and MRI scanner as covariates.

| Performance variable | Whole hippocampus | | Anterior hippocampus | | Posterior hippocampus | | Posterior/Anterior hippocampus ratio | | |  |
| --- | --- | --- | --- | --- | --- | --- | --- | --- | --- | --- |
|  | r | p | r | p | r | p | | r | p | |
|  |  |  |  |  |  |  | |  |  | |
| **Scene construction** | | | | | | | | | | |
| Experiential index | 0.16 | 0.38 | 0.21 | 0.22 | 0.083 | 0.77 | | -0.1 | 0.77 | |
| Spatial references | 0.21 | 0.22 | 0.23 | 0.22 | 0.16 | 0.38 | | -0.027 | 0.87 | |
| Entities present | 0.058 | 0.77 | 0.028 | 0.87 | 0.072 | 0.77 | | 0.064 | 0.77 | |
| Sensory descriptions | 0.16 | 0.38 | 0.19 | 0.28 | 0.096 | 0.77 | | -0.066 | 0.77 | |
| Thoughts/emotions/actions | -0.03 | 0.87 | 0.007 | 0.94 | -0.055 | 0.77 | | -0.076 | 0.77 | |
| Spatial coherence index | 0.014 | 0.93 | 0.056 | 0.77 | -0.026 | 0.87 | | -0.097 | 0.77 | |
|  |  |  |  |  |  |  | |  |  | |
| **Autobiographical interview** | | | | | | | | | | |
| Internal details | -0.035 | 0.98 | 0.029 | 0.98 | -0.082 | 0.91 | | -0.13 | 0.89 | |
| External details | 0.015 | 0.98 | -0.052 | 0.94 | 0.069 | 0.94 | | 0.12 | 0.89 | |
| Internal events | -0.098 | 0.91 | -0.049 | 0.94 | -0.12 | 0.89 | | -0.10 | 0.91 | |
| Internal time | 0.049 | 0.94 | 0.062 | 0.94 | 0.029 | 0.98 | | -0.034 | 0.98 | |
| Internal place | -0.003 | 0.98 | -0.002 | 0.98 | -0.004 | 0.98 | | -0.004 | 0.98 | |
| Internal perceptual | 0.066 | 0.94 | 0.11 | 0.89 | 0.013 | 0.98 | | -0.086 | 0.91 | |
| Internal thoughts/emotions | -0.074 | 0.94 | -0.006 | 0.98 | -0.12 | 0.89 | | -0.13 | 0.89 | |
| Vividness rating | -0.18 | 0.89 | -0.086 | 0.91 | -0.22 | 0.89 | | -0.18 | 0.89 | |
|  |  |  |  |  |  |  | |  |  | |
| **Future thinking** | | | | | | | | | | |
| Experiential index | 0.23 | 0.12 | 0.24 | 0.12 | 0.17 | 0.34 | | -0.03 | 0.88 | |
| Spatial references | 0.25 | 0.12 | 0.23 | 0.12 | 0.22 | 0.12 | | 0.051 | 0.88 | |
| Entities present | 0.12 | 0.64 | 0.11 | 0.64 | 0.10 | 0.64 | | 0.008 | 0.95 | |
| Sensory descriptions | 0.081 | 0.72 | 0.12 | 0.64 | 0.033 | 0.88 | | -0.079 | 0.72 | |
| Thoughts/emotions/actions | 0.036 | 0.88 | 0.037 | 0.88 | 0.028 | 0.88 | | -0.006 | 0.95 | |
| Spatial coherence index | 0.062 | 0.85 | 0.11 | 0.64 | 0.012 | 0.95 | | -0.098 | 0.64 | |
|  |  |  |  |  |  |  | |  |  | |
| **Navigation** | | | | | | | | | | |
| Overall navigation score | 0.044 | 0.97 | 0.079 | 0.97 | 0.005 | 0.97 | | -0.066 | 0.97 | |
| Movie clip recognition | -0.009 | 0.97 | -0.045 | 0.97 | 0.024 | 0.97 | | 0.058 | 0.97 | |
| Scene recognition | -0.12 | 0.97 | -0.10 | 0.97 | -0.12 | 0.97 | | -0.043 | 0.97 | |
| Proximity judgements | 0.16 | 0.97 | 0.12 | 0.97 | 0.16 | 0.97 | | 0.059 | 0.97 | |
| Route knowledge | 0.015 | 0.97 | 0.015 | 0.97 | 0.012 | 0.97 | | 0.009 | 0.97 | |
| Sketch map | 0.050 | 0.97 | 0.091 | 0.97 | 0.004 | 0.97 | | -0.079 | 0.97 | |
|  |  |  |  |  |  |  | |  |  | |

Note. P values are Benjamini-Hochberg false discovery rate corrected at p < 0.05.

**Table S8.** Details of the groups created for each task when dividing by median performance.

| Performance variable | Median performance | N (low performance) | N (high performance) |
| --- | --- | --- | --- |
|  |  |  |  |
| **Scene construction** |  |  |  |
| Experiential index | 41.2 | 109 | 108 |
| Spatial references | 3.29 | 112 | 105 |
| Entities present | 9.71 | 112 | 105 |
| Sensory descriptions | 12.14 | 112 | 105 |
| Thoughts/emotions/actions | 3.14 | 114 | 103 |
| Spatial coherence index | 3.0 | 112 | 105 |
|  |  |  |  |
| **Autobiographical interview** | | | |
| Internal details | 23.2 | 110 | 107 |
| External details | 4.6 | 111 | 106 |
| Internal events | 10.6 | 111 | 106 |
| Internal time | 1.4 | 128 | 89 |
| Internal place | 2.2 | 123 | 94 |
| Internal perceptual | 5.4 | 109 | 108 |
| Internal thoughts/emotions | 3.2 | 111 | 106 |
| Vividness rating | 4.6 | 114 | 103 |
|  |  |  |  |
| **Future thinking** |  |  |  |
| Experiential index | 39.8 | 111 | 106 |
| Spatial references | 2.3 | 123 | 94 |
| Entities present | 10.0 | 114 | 103 |
| Sensory descriptions | 8.67 | 117 | 100 |
| Thoughts/emotions/actions | 5.0 | 116 | 101 |
| Spatial coherence index | 2.67 | 115 | 102 |
|  |  |  |  |
| **Navigation** |  |  |  |
| Overall navigation score | 144.0 | 109 | 108 |
| Scene recognition | 30.0 | 146 | 71 |
| Proximity judgements | 8.0 | 159 | 58 |
| Route knowledge | 11.0 | 114 | 103 |
| Sketch map | 81 | 111 | 106 |
|  |  |  |  |

**Table S9.** Comparison of hippocampal grey matter volumes when dividing the sample into two groups determined by their median performance on each cognitive task, with age, gender, total intracranial volume and MRI scanner included as covariates.

| Performance variable | Whole hippocampus | | Anterior hippocampus | | Posterior hippocampus | | Posterior/Anterior hippocampus ratio | | |  |
| --- | --- | --- | --- | --- | --- | --- | --- | --- | --- | --- |
|  | F | p | F | p | F | p | | F | p | |
|  |  |  |  |  |  |  | |  |  | |
| **Scene construction** | | | | | | | | | | |
| Experiential index | 0.85 | 0.70 | 0.48 | 0.83 | 0.88 | 0.70 | | 0.051 | 0.99 | |
| Spatial references | 1.09 | 0.70 | 0.95 | 0.70 | 0.79 | 0.70 | | 0.002 | 0.99 | |
| Entities present | 0.043 | 0.99 | 1.06 | 0.70 | 0.35 | 0.83 | | 2.93 | 0.70 | |
| Sensory descriptions | 0.88 | 0.70 | 0.013 | 0.99 | 2.27 | 0.70 | | 2.45 | 0.70 | |
| Thoughts/emotions/actions | 1.30 | 0.70 | 1.26 | 0.70 | 0.85 | 0.70 | | 0.020 | 0.99 | |
| Spatial coherence index | 0.031 | 0.99 | 0.11 | 0.99 | 0.00 | 0.99 | | 0.39 | 0.83 | |
|  |  |  |  |  |  |  | |  |  | |
| **Autobiographical interview** | | | | | | | | | | |
| Internal details | 0.96 | 0.81 | 0.007 | 0.95 | 2.59 | 0.70 | | 4.18 | 0.62 | |
| External details | 0.53 | 0.81 | 3.65 | 0.62 | 0.26 | 0.81 | | 5.81 | 0.54 | |
| Internal events | 0.76 | 0.81 | 0.005 | 0.95 | 2.06 | 0.78 | | 2.69 | 0.70 | |
| Internal time | 0.27 | 0.81 | 0.29 | 0.81 | 0.16 | 0.88 | | 0.004 | 0.95 | |
| Internal place | 0.13 | 0.89 | 0.009 | 0.95 | 0.50 | 0.81 | | 1.17 | 0.81 | |
| Internal perceptual | 0.071 | 0.94 | 0.042 | 0.95 | 0.42 | 0.81 | | 1.44 | 0.81 | |
| Internal thoughts/emotions | 0.63 | 0.81 | 0.27 | 0.81 | 0.77 | 0.81 | | 0.58 | 0.81 | |
| Vividness ratings | 1.56 | 0.81 | 0.69 | 0.81 | 1.90 | 0.78 | | 0.57 | 0.81 | |
|  |  |  |  |  |  |  | |  |  | |
| **Future thinking** | | | | | | | | | | |
| Experiential index | 0.11 | 0.96 | 0.17 | 0.96 | 0.033 | 0.96 | | 0.15 | 0.96 | |
| Spatial references | 1.50 | 0.96 | 2.71 | 0.96 | 0.34 | 0.96 | | 0.58 | 0.96 | |
| Entities present | 0.009 | 0.96 | 0.055 | 0.96 | 0.003 | 0.96 | | 0.13 | 0.96 | |
| Sensory descriptions | 0.062 | 0.96 | 1.58 | 0.96 | 0.54 | 0.96 | | 3.97 | 0.96 | |
| Thoughts/emotions/actions | 0.17 | 0.96 | 0.11 | 0.96 | 1.03 | 0.96 | | 1.87 | 0.96 | |
| Spatial coherence index | 0.005 | 0.96 | 0.006 | 0.96 | 0.039 | 0.96 | | 0.020 | 0.96 | |
|  |  |  |  |  |  |  | |  |  | |
| **Navigation** | | | | | | | | | | |
| Overall navigation score | 0.11 | 0.96 | 0.49 | 0.96 | 0.008 | 0.96 | | 0.74 | 0.96 | |
| Scene recognition | 0.098 | 0.96 | 0.23 | 0.96 | 0.009 | 0.96 | | 0.19 | 0.96 | |
| Proximity judgements | 0.079 | 0.96 | 0.046 | 0.96 | 0.081 | 0.96 | | 0.003 | 0.96 | |
| Route knowledge | 0.72 | 0.96 | 1.76 | 0.96 | 0.052 | 0.96 | | 1.12 | 0.96 | |
| Sketch map | 0.11 | 0.96 | 0.29 | 0.96 | 0.007 | 0.96 | | 0.21 | 0.96 | |
|  |  |  |  |  |  |  | |  |  | |

Note. P values are Benjamini-Hochberg false discovery rate corrected at p < 0.05.

**Table S10.** Partial correlations between task performance and hippocampal grey matter volume in the low performing participants only (as determined by a median split for each task) with age, gender, total intracranial volume and MRI scanner as covariates.

| Performance variable | Whole hippocampus | | Anterior hippocampus | | Posterior hippocampus | | Posterior/Anterior hippocampus ratio | | |  |
| --- | --- | --- | --- | --- | --- | --- | --- | --- | --- | --- |
|  | r | p | r | p | r | p | | r | p | |
|  |  |  |  |  |  |  | |  |  | |
| **Scene construction** | | | | | | | | | | |
| Experiential index | 0.002 | 0.98 | 0.059 | 0.98 | -0.054 | 0.98 | | 0.10 | 0.96 | |
| Spatial references | 0.019 | 0.98 | -0.003 | 0.98 | 0.036 | 0.98 | | 0.052 | 0.98 | |
| Entities present | 0.013 | 0.98 | -0.005 | 0.98 | 0.027 | 0.98 | | 0.042 | 0.98 | |
| Sensory descriptions | 0.17 | 0.88 | 0.18 | 0.88 | 0.14 | 0.96 | | -0.008 | 0.98 | |
| Thoughts/emotions/actions | -0.057 | 0.98 | -0.11 | 0.96 | 0.008 | 0.98 | | 0.11 | 0.96 | |
| Spatial coherence index | 0.053 | 0.98 | 0.097 | 0.96 | -0.007 | 0.98 | | -0.10 | 0.96 | |
|  |  |  |  |  |  |  | |  |  | |
| **Autobiographical interview** | | | | | | | | | | |
| Internal details | -0.032 | 0.91 | -0.022 | 0.96 | -0.033 | 0.91 | | -0.004 | 0.97 | |
| External details | -0.082 | 0.90 | -0.047 | 0.90 | -0.10 | 0.90 | | -0.065 | 0.90 | |
| Internal events | -0.072 | 0.90 | -0.020 | 0.96 | -0.11 | 0.90 | | -0.11 | 0.90 | |
| Internal time | 0.047 | 0.90 | 0.006 | 0.97 | 0.073 | 0.90 | | 0.064 | 0.90 | |
| Internal place | -0.049 | 0.90 | -0.075 | 0.90 | -0.014 | 0.97 | | 0.042 | 0.90 | |
| Internal perceptual | -0.054 | 0.90 | -0.099 | 0.90 | 0.010 | 0.97 | | 0.13 | 0.90 | |
| Internal thoughts/emotions | 0.047 | 0.90 | -0.034 | 0.91 | 0.12 | 0.90 | | 0.16 | 0.90 | |
| Vividness ratings | -0.11 | 0.90 | -0.077 | 0.90 | -0.11 | 0.90 | | -0.067 | 0.90 | |
|  |  |  |  |  |  |  | |  |  | |
| **Future thinking** | | | | | | | | | | |
| Experiential index | 0.075 | 0.99 | 0.14 | 0.99 | 0.001 | 0.99 | | -0.11 | 0.99 | |
| Spatial references | 0.032 | 0.99 | 0.008 | 0.99 | 0.047 | 0.99 | | 0.063 | 0.99 | |
| Entities present | -0.003 | 0.99 | 0.019 | 0.99 | -0.023 | 0.99 | | -0.039 | 0.99 | |
| Sensory descriptions | 0.056 | 0.99 | 0.061 | 0.99 | 0.041 | 0.99 | | -0.010 | 0.99 | |
| Thoughts/emotions/actions | -0.069 | 0.99 | -0.008 | 0.99 | -0.11 | 0.99 | | -0.14 | 0.99 | |
| Spatial coherence index | -0.049 | 0.99 | 0.040 | 0.99 | -0.12 | 0.99 | | -0.19 | 0.99 | |
|  |  |  |  |  |  |  | |  |  | |
| **Navigation** | | | | | | | | | | |
| Overall navigation score | 0.29 | 0.06 | 0.24 | 0.065 | 0.27 | 0.06 | | 0.067 | 0.60 | |
| Scene recognition | -0.077 | 0.55 | -0.094 | 0.49 | -0.044 | 0.67 | | 0.055 | 0.60 | |
| Proximity judgements | 0.082 | 0.52 | 0.054 | 0.60 | 0.094 | 0.48 | | 0.057 | 0.60 | |
| Route knowledge | 0.18 | 0.19 | 0.16 | 0.24 | 0.15 | 0.24 | | 0.032 | 0.78 | |
| Sketch map | 0.25 | 0.065 | 0.22 | 0.084 | 0.21 | 0.090 | | 0.020 | 0.84 | |
|  |  |  |  |  |  |  | |  |  | |

Note. P values are Benjamini-Hochberg false discovery rate corrected at p < 0.05.

**Table S11.** Partial correlations between performance and hippocampal grey matter volume in the high performing participants only (as determined by a median split for each task) with age, gender, total intracranial volume and MRI scanner as covariates.

| Performance variable | Whole hippocampus | | Anterior hippocampus | | Posterior hippocampus | | Posterior/Anterior hippocampus ratio | | |  |
| --- | --- | --- | --- | --- | --- | --- | --- | --- | --- | --- |
|  | r | p | r | p | r | p | | r | p | |
|  |  |  |  |  |  |  | |  |  | |
| **Scene construction** | | | | | | | | | | |
| Experiential index | -0.019 | 0.99 | -0.001 | 0.99 | -0.032 | 0.99 | | -0.033 | 0.99 | |
| Spatial references | 0.057 | 0.99 | 0.067 | 0.99 | 0.036 | 0.99 | | -0.027 | 0.99 | |
| Entities present | -0.017 | 0.99 | -0.021 | 0.99 | -0.009 | 0.99 | | 0.012 | 0.99 | |
| Sensory descriptions | 0.078 | 0.99 | 0.066 | 0.99 | 0.071 | 0.99 | | -0.001 | 0.99 | |
| Thoughts/emotions/actions | 0.096 | 0.99 | 0.062 | 0.99 | 0.11 | 0.99 | | 0.072 | 0.99 | |
| Spatial coherence index | 0.014 | 0.99 | -0.072 | 0.99 | 0.080 | 0.99 | | 0.18 | 0.99 | |
|  |  |  |  |  |  |  | |  |  | |
| **Autobiographical interview** | | | | | | | | | | |
| Internal details | 0.0 | 1.0 | 0.004 | 1.0 | -0.003 | 1.0 | | -0.003 | 1.0 | |
| External details | 0.11 | 1.0 | 0.12 | 1.0 | 0.076 | 1.0 | | -0.023 | 1.0 | |
| Internal events | -0.016 | 1.0 | -0.027 | 1.0 | -0.001 | 1.0 | | 0.029 | 1.0 | |
| Internal time | -0.067 | 1.0 | 0.004 | 1.0 | -0.12 | 1.0 | | -0.16 | 1.0 | |
| Internal place | -0.087 | 1.0 | -0.13 | 1.0 | -0.037 | 1.0 | | 0.10 | 1.0 | |
| Internal perceptual | 0.047 | 1.0 | 0.12 | 1.0 | -0.018 | 1.0 | | -0.13 | 1.0 | |
| Internal thoughts/emotions | -0.040 | 1.0 | 0.005 | 1.0 | -0.072 | 1.0 | | -0.11 | 1.0 | |
| Vividness ratings | -0.093 | 1.0 | -0.087 | 1.0 | -0.079 | 1.0 | | -0.013 | 1.0 | |
|  |  |  |  |  |  |  | |  |  | |
| **Future thinking** | | | | | | | | | | |
| Experiential index | -0.027 | 0.91 | 0.017 | 0.91 | -0.061 | 0.91 | | -0.082 | 0.91 | |
| Spatial references | -0.012 | 0.91 | 0.035 | 0.91 | -0.054 | 0.91 | | -0.11 | 0.91 | |
| Entities present | -0.012 | 0.91 | -0.064 | 0.91 | 0.040 | 0.91 | | 0.10 | 0.91 | |
| Sensory descriptions | -0.11 | 0.91 | -0.16 | 0.91 | -0.047 | 0.91 | | 0.097 | 0.91 | |
| Thoughts/emotions/actions | -0.099 | 0.91 | -0.089 | 0.91 | -0.088 | 0.91 | | -0.022 | 0.91 | |
| Spatial coherence index | -0.066 | 0.91 | -0.073 | 0.91 | -0.046 | 0.91 | | 0.023 | 0.91 | |
|  |  |  |  |  |  |  | |  |  | |
| **Navigation** | | | | | | | | | | |
| Overall navigation score | -0.071 | 0.69 | 0.039 | 0.74 | -0.15 | 0.33 | | -0.22 | 0.24 | |
| Scene recognition | 0.11 | 0.63 | 0.16 | 0.40 | 0.038 | 0.76 | | -0.14 | 0.47 | |
| Proximity judgements | 0.24 | 0.33 | 0.21 | 0.33 | 0.20 | 0.33 | | 0.06 | 0.73 | |
| Route knowledge | 0.044 | 0.73 | 0.15 | 0.33 | -0.06 | 0.73 | | -0.21 | 0.26 | |
| Sketch map | -0.082 | 0.63 | 0.048 | 0.73 | -0.18 | 0.33 | | -0.27 | 0.12 | |
|  |  |  |  |  |  |  | |  |  | |

Note. P values are Benjamini-Hochberg false discovery rate corrected at p < 0.05.

**Table S12.** Details of the groups created for each task when taking only the best and worst performers.

| Performance variable | Worst performers maximum | Best performers minimum | N (worst performers) | N (best performers) |
| --- | --- | --- | --- | --- |
|  |  |  |  |  |
| **Scene construction** |  |  |  |  |
| Experiential index | 32.66 | 48.49 | 20 | 20 |
| Spatial references | 1.57 | 5.57 | 21 | 22 |
| Entities present | 6.29 | 13.86 | 20 | 20 |
| Sensory descriptions | 7.71 | 17.0 | 21 | 21 |
| Thoughts/emotions/actions | 1.29 | 6.14 | 19 | 18 |
| Spatial coherence index | 0.71 | 5.0 | 23 | 25 |
|  |  |  |  |  |
| **Autobiographical interview** | | | | |
| Internal details | 15.20 | 34.60 | 22 | 21 |
| External details | 1.80 | 10.20 | 21 | 21 |
| Internal events | 6.20 | 16.60 | 21 | 21 |
| Internal time | 2.20 | 10.0 | 18 | 15 |
| Internal place | 1.40 | 3.0 | 26 | 32 |
| Internal perceptual | 0.40 | 2.60 | 20 | 21 |
| Internal thoughts/emotions | 1.20 | 6.0 | 19 | 18 |
| Vividness rating | 3.40 | 5.60 | 19 | 20 |
|  |  |  |  |  |
| **Future thinking** |  |  |  |  |
| Experiential index | 30.13 | 47.33 | 21 | 21 |
| Spatial references | 0.67 | 4.33 | 32 | 30 |
| Entities present | 6.67 | 14.67 | 23 | 22 |
| Sensory descriptions | 4.33 | 13.33 | 21 | 21 |
| Thoughts/emotions/actions | 2.33 | 8.33 | 22 | 22 |
| Spatial coherence index | 0.0 | 5.0 | 23 | 29 |
|  |  |  |  |  |
| **Navigation** |  |  |  |  |
| Overall navigation score | 95.0 | 191.0 | 21 | 21 |
| Scene recognition | 27.0 | 32.0 | 34 | 28 |
| Proximity judgements | 5.0 | 10.0 | 18 | 13 |
| Route knowledge | 6.0 | 19.0 | 23 | 22 |
| Sketch map | 39.0 | 121.0 | 21 | 21 |
|  |  |  |  |  |

**Table S13.** Comparison of hippocampal grey matter volumes when taking the best and worst performing participants for task, with age, gender, total intracranial volume and MRI scanner included as covariates.

| Performance variable | Whole hippocampus | | Anterior hippocampus | | Posterior hippocampus | | Posterior/Anterior hippocampus ratio | | |  |
| --- | --- | --- | --- | --- | --- | --- | --- | --- | --- | --- |
|  | F | p | F | p | F | p | | F | p | |
|  |  |  |  |  |  |  | |  |  | |
| **Scene construction** | | | | | | | | | | |
| Experiential index | 0.092 | 0.97 | 0.36 | 0.97 | 0.0 | 1.0 | | 0.39 | 0.97 | |
| Spatial references | 0.69 | 0.97 | 0.15 | 0.97 | 1.24 | 0.97 | | 1.34 | 0.97 | |
| Entities present | 0.0 | 1.0 | 0.38 | 0.97 | 0.37 | 0.97 | | 1.94 | 0.97 | |
| Sensory descriptions | 0.47 | 0.97 | 0.63 | 0.97 | 0.27 | 0.97 | | 0.006 | 1.0 | |
| Thoughts/emotions/actions | 0.014 | 1.0 | 0.21 | 0.97 | 0.49 | 0.97 | | 1.29 | 0.97 | |
| Spatial coherence index | 0.22 | 0.97 | 0.34 | 0.97 | 0.075 | 0.97 | | 0.058 | 0.97 | |
|  |  |  |  |  |  |  | |  |  | |
| **Autobiographical interview** | | | | | | | | | | |
| Internal details | 4.09 | 0.80 | 1.17 | 0.88 | 6.04 | 0.61 | | 2.44 | 0.83 | |
| External details | 0.73 | 0.88 | 1.36 | 0.88 | 0.089 | 0.88 | | 0.69 | 0.88 | |
| Internal events | 1.56 | 0.88 | 0.30 | 0.88 | 2.62 | 0.83 | | 1.67 | 0.88 | |
| Internal time | 0.001 | 0.98 | 0.053 | 0.90 | 0.035 | 0.91 | | 0.23 | 0.88 | |
| Internal place | 0.48 | 0.88 | 0.10 | 0.88 | 0.85 | 0.88 | | 0.58 | 0.88 | |
| Internal perceptual | 0.18 | 0.88 | 0.13 | 0.88 | 0.17 | 0.88 | | 0.092 | 0.88 | |
| Internal thoughts/emotions | 0.51 | 0.88 | 0.87 | 0.88 | 0.17 | 0.88 | | 0.43 | 0.88 | |
| Vividness ratings | 2.89 | 0.98 | 1.85 | 0.88 | 2.42 | 0.83 | | 0.097 | 0.88 | |
|  |  |  |  |  |  |  | |  |  | |
| **Future thinking** | | | | | | | | | | |
| Experiential index | 0.17 | 0.96 | 0.80 | 0.96 | 0.001 | 0.98 | | 0.85 | 0.96 | |
| Spatial references | 0.74 | 0.96 | 3.06 | 0.96 | 0.001 | 0.98 | | 1.75 | 0.96 | |
| Entities present | 0.20 | 0.96 | 0.029 | 0.98 | 0.37 | 0.96 | | 0.44 | 0.96 | |
| Sensory descriptions | 0.003 | 0.98 | 0.074 | 0.96 | 0.12 | 0.96 | | 0.43 | 0.96 | |
| Thoughts/emotions/actions | 0.30 | 0.96 | 0.44 | 0.96 | 0.12 | 0.96 | | 0.062 | 0.96 | |
| Spatial coherence index | 0.72 | 0.96 | 0.24 | 0.96 | 1.02 | 0.96 | | 0.50 | 0.96 | |
|  |  |  |  |  |  |  | |  |  | |
| **Navigation** | | | | | | | | | | |
| Overall navigation score | 0.88 | 0.47 | 4.51 | 0.09 | 0.071 | 0.79 | | 8.39 | 0.06 | |
| Scene recognition | 0.58 | 0.56 | 0.93 | 0.47 | 0.21 | 0.72 | | 0.25 | 0.72 | |
| Proximity judgements | 5.38 | 0.07 | 2.57 | 0.22 | 6.19 | 0.067 | | 1.84 | 0.32 | |
| Route knowledge | 7.34 | 0.07 | 6.78 | 0.065 | 4.30 | 0.09 | | 6.15 | 0.067 | |
| Sketch map | 0.99 | 0.47 | 5.18 | 0.073 | 0.098 | 0.79 | | 10.01 | 0.06 | |
|  |  |  |  |  |  |  | |  |  | |

Note. P values are Benjamini-Hochberg false discovery rate corrected at p < 0.05.

**Table S14.** Partial correlations between task performance and the interaction between hippocampal grey matter volume and self-reported ability (measured by the Plymouth Sensory Imagery Questionnaire for scene construction, autobiographical memory and future thinking and the Santa Barbara Sense of Direction Scale for navigation), with age, gender, total intracranial volume and MRI scanner included as covariates in all analyses, as well as the additional covariates of the relevant questionnaire and hippocampal volume measurement.

| Performance variable | Whole hippocampus | | Anterior hippocampus | | Posterior hippocampus | | Posterior/Anterior hippocampus ratio | |  |
| --- | --- | --- | --- | --- | --- | --- | --- | --- | --- |
|  | r | p | r | p | r | p | r | p | |
|  |  |  |  |  |  |  |  |  | |
| **Scene construction** | | | | | | | | | |
| Experiential index | 0.044 | 0.94 | 0.054 | 0.94 | 0.029 | 0.94 | -0.023 | 0.94 | |
| Spatial references | -0.029 | 0.94 | -0.01 | 0.94 | -0.047 | 0.94 | -0.022 | 0.94 | |
| Entities present | 0.002 | 0.97 | 0.009 | 0.94 | -0.017 | 0.94 | -0.038 | 0.94 | |
| Sensory descriptions | -0.076 | 0.94 | -0.057 | 0.94 | -0.078 | 0.94 | -0.008 | 0.94 | |
| Thoughts/emotions/actions | 0.055 | 0.94 | 0.052 | 0.94 | 0.047 | 0.94 | -0.021 | 0.94 | |
| Spatial coherence index | 0.015 | 0.94 | 0.019 | 0.94 | 0.01 | 0.94 | -0.021 | 0.94 | |
|  |  |  |  |  |  |  |  |  | |
| **Autobiographical interview** | | | | | | | | | |
| Internal details | 0.014 | 0.87 | 0.018 | 0.87 | 0.017 | 0.87 | -0.022 | 0.87 | |
| External details | 0.084 | 0.78 | 0.11 | 0.50 | 0.033 | 0.87 | -0.12 | 0.49 | |
| Internal events | 0.029 | 0.87 | 0.033 | 0.87 | 0.031 | 0.87 | -0.032 | 0.87 | |
| Internal time | -0.052 | 0.87 | -0.043 | 0.87 | -0.054 | 0.87 | 0.001 | 0.99 | |
| Internal place | 0.029 | 0.87 | 0.075 | 0.87 | -0.027 | 0.87 | -0.14 | 0.29 | |
| Internal perceptual | -0.034 | 0.87 | -0.015 | 0.87 | -0.042 | 0.87 | -0.028 | 0.87 | |
| Internal thoughts/emotions | 0.067 | 0.87 | 0.02 | 0.87 | 0.11 | 0.50 | 0.086 | 0.78 | |
| Vividness ratings | 0.22 | 0.016* | 0.18 | 0.096 | 0.24 | 0.013* | 0.026 | 0.87 | |
|  |  |  |  |  |  |  |  |  | |
| **Future thinking** | | | | | | | | | |
| Experiential index | -0.018 | 0.90 | 0.012 | 0.91 | -0.041 | 0.82 | -0.041 | 0.82 | |
| Spatial references | -0.07 | 0.82 | -0.028 | 0.82 | -0.1 | 0.82 | -0.051 | 0.82 | |
| Entities present | -0.062 | 0.82 | -0.05 | 0.82 | -0.068 | 0.82 | -0.007 | 0.91 | |
| Sensory descriptions | -0.12 | 0.82 | -0.09 | 0.82 | -0.13 | 0.82 | -0.033 | 0.82 | |
| Thoughts/emotions/actions | 0.066 | 0.82 | 0.076 | 0.82 | 0.042 | 0.82 | -0.072 | 0.82 | |
| Spatial coherence index | 0.03 | 0.82 | 0.045 | 0.82 | 0.011 | 0.91 | -0.03 | 0.82 | |
|  |  |  |  |  |  |  |  |  | |
| **Navigation** | | | | | | | | | |
| Overall navigation score | 0.049 | 0.85 | 0.026 | 0.85 | 0.067 | 0.72 | 0.035 | 0.85 | |
| Clip recognition | 0.084 | 0.72 | 0.084 | 0.72 | 0.071 | 0.72 | -0.04 | 0.85 | |
| Scene recognition | -0.11 | 0.72 | -0.088 | 0.72 | -0.12 | 0.72 | -0.02 | 0.88 | |
| Proximity judgements | 0.1 | 0.72 | 0.074 | 0.72 | 0.11 | 0.72 | 0.047 | 0.85 | |
| Route knowledge | 0.002 | 0.98 | -0.012 | 0.90 | 0.016 | 0.89 | 0.028 | 0.85 | |
| Sketch map | 0.056 | 0.84 | 0.031 | 0.85 | 0.075 | 0.72 | 0.037 | 0.85 | |
|  |  |  |  |  |  |  |  |  | |

Note. P values are Benjamini-Hochberg false discovery rate corrected at p < 0.05. * = p < 0.05.

**Table S15**. Details of the principal component analysis on the four main outcome measures.

| Cognitive task | Component weighting |
| --- | --- |
|  |  |
| Scene construction experiential index | 0.90 |
| Future thinking experiential index | 0.87 |
| Autobiographical interview internal details | 0.66 |
| Navigation | 0.49 |
|  |  |

Note. The factor scores for each participant were extracted using the Anderson-Rubin method. These were included in a VBM analysis with age, gender, scanner and total intracranial volume as covariates, finding no significant relationships with hippocampal volume both at the whole brain level (p < 0.05 FWE corrected) and when using the anatomical hippocampal masks (p < 0.05 FWE corrected for each mask). Performing partial correlations between the extracted hippocampal grey matter volumes and the factor scores also identified no significant relationships (whole hippocampal volume: r = 0.032, p _FDR corrected_ = 0.85; anterior hippocampal volume: r = 0.073, p _FDR corrected_ = 0.58; posterior hippocampal volume: r = -0.012, p _FDR corrected_ = 0.86; posterior/anterior hippocampal volume ratio: r = -0.091, p _FDR corrected_ = 0.58).

**Table S16**. Proportion of variance explained by each principal component of the sub-measures principal component analysis.

| PCA Component | Variance explained |
| --- | --- |
|  |  |
| C1. Content scenes and future | 19.11 |
| C2. Navigation | 11.67 |
| C3. Spatial coherence | 9.88 |
| C4. Thoughts/emotions | 9.42 |
| C5. Autobiographical memory | 9.21 |
| C6. Time | 5.73 |
|  |  |

**Table S17.** Details of the principal component analysis conducted on all 21 task sub-measures.

| Sub-measure | Content scenes and future | Navigation | Spatial  coherence | Thoughts/  emotions | Autobiographical memory | Time |
| --- | --- | --- | --- | --- | --- | --- |
|  |  |  |  |  |  |  |
| Future thinking entities present | 0.84 |  |  |  |  |  |
| Future thinking spatial references | 0.78 |  |  |  |  |  |
| Future thinking sensory descriptions | 0.76 |  |  |  |  |  |
| Scene construction sensory descriptions | 0.75 |  |  |  |  |  |
| Scene construction entities present | 0.72 |  |  | 0.41 |  |  |
| Scene construction spatial references | 0.70 |  |  |  |  |  |
| Navigation route knowledge |  | 0.84 |  |  |  |  |
| Navigation sketch map |  | 0.83 |  |  |  |  |
| Navigation movie clip recognition |  | 0.64 |  |  |  |  |
| Navigation scene recognition |  | 0.59 |  |  |  |  |
| Navigation proximity judgements |  | 0.41 |  |  |  |  |
| Scene construction spatial coherence index |  |  | 0.89 |  |  |  |
| Future thinking spatial coherence index |  |  | 0.87 |  |  |  |
| Autobiographical memory vividness |  |  | 0.61 |  |  | 0.38 |
| Scene construction thoughts/emotions/actions |  |  |  | 0.84 |  |  |
| Future thinking thoughts/emotions/actions |  |  |  | 0.76 |  |  |
| Autobiographical memory internal thoughts/emotions |  |  |  | 0.49 | 0.57 |  |
| Autobiographical memory internal events |  |  |  |  | 0.79 |  |
| Autobiographical memory internal perceptual |  |  |  |  | 0.65 |  |
| Autobiographical memory internal place |  |  |  |  | 0.57 | 0.35 |
| Autobiographical memory internal time |  |  |  |  |  | 0.73 |
|  |  |  |  |  |  |  |

Note. Task order is for display purposes only. Only values over 0.3 are reported for ease of viewing.

**Table S18.** Partial correlations between the sub-measures principal components and hippocampal grey matter volume with age, gender, total intracranial volume and MRI scanner as covariates.

| PCA component | Whole hippocampus | | Anterior hippocampus | | Posterior hippocampus | | Posterior/Anterior hippocampus ratio | |
| --- | --- | --- | --- | --- | --- | --- | --- | --- |
|  | r | p | r | p | r | p | r | p |
|  | | | | | | | | |
| C1. Content scenes and future | 0.041 | 0.78 | 0.048 | 0.78 | 0.027 | 0.88 | -0.009 | 0.97 |
| C2. Navigation | 0.12 | 0.65 | 0.13 | 0.65 | 0.085 | 0.66 | -0.029 | 0.88 |
| C3. Spatial coherence | -0.044 | 0.78 | -0.021 | 0.91 | -0.056 | 0.77 | -0.055 | 0.77 |
| C4. Thoughts/emotions | -0.052 | 0.77 | -0.084 | 0.66 | -0.011 | 0.97 | 0.060 | 0.77 |
| C5. Autobiographical memory | -0.052 | 0.77 | 0.002 | 0.98 | -0.091 | 0.66 | -0.12 | 0.65 |
| C6. Time | -0.096 | 0.66 | -0.097 | 0.66 | -0.075 | 0.75 | 0.006 | 0.97 |
|  |  |  |  |  |  |  |  |  |

Note. P values are Benjamini-Hochberg false discovery rate corrected at p < 0.05.


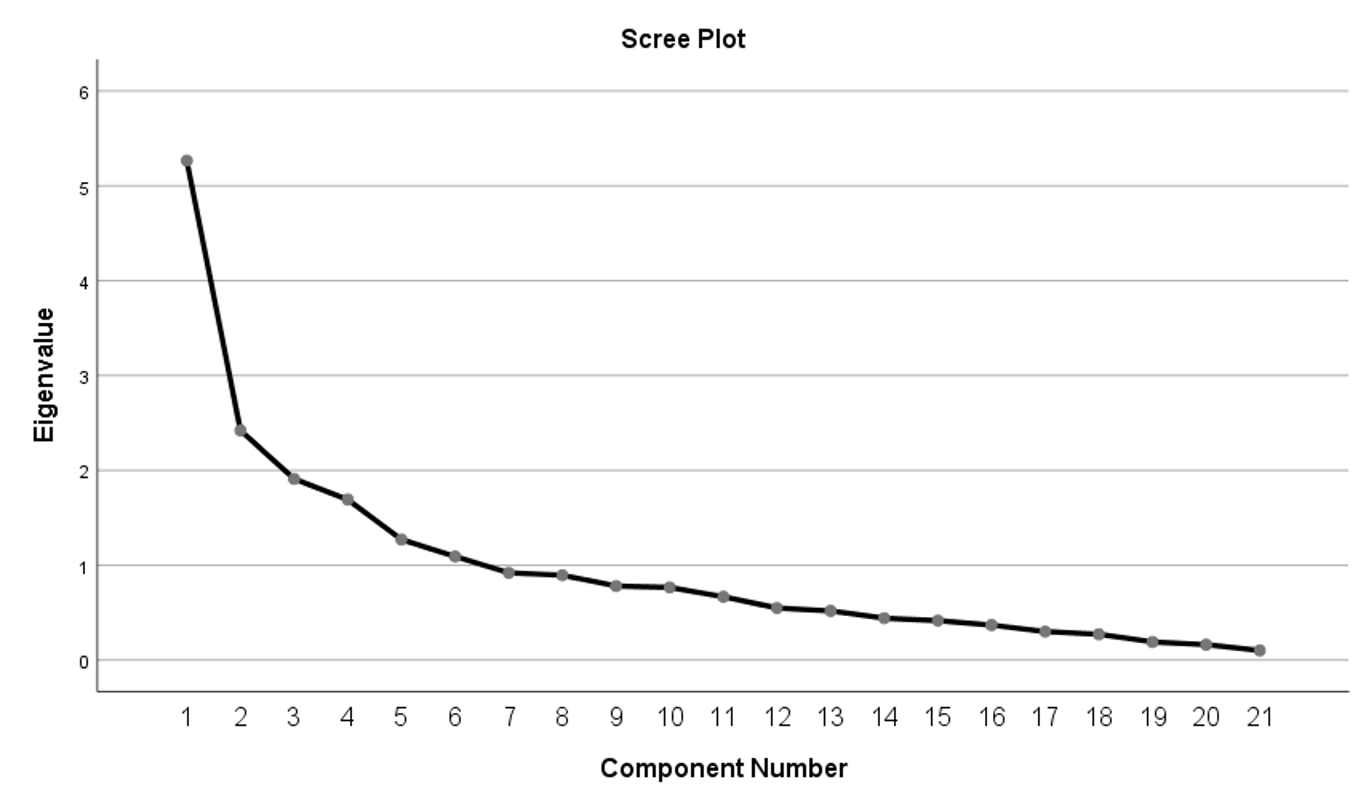


**Figure S1**. Scree plot of the principal component analysis performed on the sub-measures.
